# Combining Multiple RNA-Seq Data Analysis Algorithms Using Machine Learning Improves Differential Isoform Expression Analysis

**DOI:** 10.3390/mps4040068

**Published:** 2021-09-27

**Authors:** Alexandros C. Dimopoulos, Konstantinos Koukoutegos, Fotis E. Psomopoulos, Panagiotis Moulos

**Affiliations:** 1 Institute for Fundamental Biomedical Research, Biomedical Sciences Research Center ‘Alexander Fleming’, Fleming 34, 16672 Vari, Greece; dimopoulos@fleming.gr or; 2 Hellenic Naval Academy, Hatzikyriakou Ave., 18539 Piraeus, Greece; 3 Institute of Applied Biosciences, Centre for Research and Technology Hellas, 6th Km Charilaou-Thermis Rd, 57001 Thessaloniki, Greece; konstantinos.koukoutegos@gmail.com (K.K.); fpsom@certh.gr (F.E.P.)

**Keywords:** RNA-sequencing, alternative splicing, machine learning, docker, shiny

## Abstract

RNA sequencing has become the standard technique for high resolution genome-wide monitoring of gene expression. As such, it often comprises the first step towards understanding complex molecular mechanisms driving various phenotypes, spanning organ development to disease genesis, monitoring and progression. An advantage of RNA sequencing is its ability to capture complex transcriptomic events such as alternative splicing which results in alternate isoform abundance. At the same time, this advantage remains algorithmically and computationally challenging, especially with the emergence of even higher resolution technologies such as single-cell RNA sequencing. Although several algorithms have been proposed for the effective detection of differential isoform expression from RNA-Seq data, no widely accepted golden standards have been established. This fact is further compounded by the significant differences in the output of different algorithms when applied on the same data. In addition, many of the proposed algorithms remain scarce and poorly maintained. Driven by these challenges, we developed a novel integrative approach that effectively combines the most widely used algorithms for differential transcript and isoform analysis using state-of-the-art machine learning techniques. We demonstrate its usability by applying it on simulated data based on several organisms, and using several performance metrics; we conclude that our strategy outperforms the application of the individual algorithms. Finally, our approach is implemented as an R Shiny application, with the underlying data analysis pipelines also available as docker containers.

## 1. Introduction

A common application of next-generation sequencing (NGS) is RNA-sequencing (RNA-Seq), which is the genome-wide monitoring of gene expression across organisms, tissues, and lately, single cells [1]. Due to its higher signal resolution, lower amounts of required input material, wider dynamic range of measurements [2] and greater range of applications, RNA-Seq has become the standard technique for such studies. In addition, the continuously dropping sequencing costs and the emergence of new technologies and less costly protocols such as Quant-Seq 3′ mRNA sequencing [3], have contributed to an ever-expanding landscape of data generation in order to characterize gene expression changes across tissues, organs, biological conditions and whole organisms. Furthermore, RNA-Seq has been deployed for a plethora of applications including but not limited to de novo transcriptome assembly [4], investigation of alternative splicing [5], discovery of fused transcripts [6], interrogation of allele-specific gene expression [7], genetic variant discovery [8] and detection of non-coding genes [9]. Therefore, it is evident that RNA-Seq has become the standard technique for experimental designs involving gene expression and often gene sequence alterations and has permanently replaced outdated techniques such as DNA microarrays [9].

Although RNA-Seq has been used for a variety of applications, its most common use remains the genome-wide gene expression monitoring across different biological conditions in the search for alternatively expressed genes, gene sets and encompassing biochemical pathways that characterize such conditions such as disease progression or organ development [10]. Therefore, commonly asked questions from RNA-Seq deal with the detection of differentially expressed transcripts across different conditions in the process of differential expression analysis. An important aspect in RNA-Seq and among its revolutionary contributions is its ability to capture alterations in gene splicing and the subsequent isoform expression. Alternative splicing is a process during gene expression that allows a single gene to code for multiple proteins [11]. This is achieved through the inclusion or exclusion of the gene’s exons during its transcription to mRNA. Alternative splicing occurs in eukaryotes and demonstrates major contributions in cellular differentiation, organism development and protein biodiversity [12]. In addition, deviations in alternative splicing have been shown to be connected to several diseases, including auto-immune syndromes [13], cardiomyopathies [14] and cancer [15,16]. It is therefore evident that proper and accurate detection of alternative splicing leading to differential isoform expression is essential to understanding not only fundamental biological mechanisms, but also disease genesis and progression.

During the past decade, serious research effort has been devoted in the development of methods for differential isoform expression analysis (DIEA) and alternative splicing (AS) while also trying to address some of its inherent biases, such as the gene-length bias (read accumulation over longer transcripts) [17] which may propagate to DIEA. This led to a series of available analysis tools which, along with the expanding data volumes, often leaves researchers confused, as not only must they extract meaningful biological outcomes from the latter but also navigate through a variety of methods, many being poorly documented or unmaintained. Furthermore, non-consistent or non-compatible outputs of many of these tools contribute to the interpretation burden by field experts, as they are based on different execution models and provide as output different and sometimes incompatible/incomparable metrics for differential expression assessments and predictions.

Finally, a quite common observation when using multiple tools for DIEA of an RNA-Seq dataset is that while the input data to each method are the same, the different tools tested usually do not reach the same conclusions, making it difficult not only to decide on the best methodology but also to aggregate the independent results towards a reliable consensus suitable for downstream analysis [18].

To tackle such problems for simple differential expression analysis at the whole gene level (i.e., not differential isoform expression), we previously developed PANDORA, a weighted *p*-value combination algorithm [19]. By combining individual test *p*-values through weights estimated from real data, PANDORA was shown to optimize the result accuracy by ensuring the best precision-sensitivity relationship relative to the rest of the tested tools and other *p*-value combination methods. Furthermore, we recently showed evidence that PANDORA is insusceptible to biases introduced by different normalization frameworks, showed improved performance when applied to low count RNA molecules, such as lncRNAs and was able to control the propagation of gene length bias into downstream enrichment analysis [20].

Driven by our previous results, we hereby extend the PANDORA concept towards DIEA in an effort to implement a unified framework to address the aforementioned algorithm compatibility and consistency issues. We argue that the approach of *p*-value combination is no longer applicable in the case of DIEA due to the absence of compatible and comparable statistical scores reported by the examined DIEA tools and we propose metaDIEA, an integration approach based on established machine learning (ML) techniques. By using ML, we were able to successfully integrate six distinct DIEA tools and demonstrate the increased performance of our approach using simulated data across different organisms. Specifically, we provide evidence that the combined approach outperforms the application of single DIEA algorithms on simulated datasets and successfully overcomes the discussed output compatibility issues by reporting a single score constructed by the ML approach. The computational pipelines behind metaDIEA are available as docker containers which are also interfaced through an R Shiny application available at https://github.com/BiodataAnalysisGroup/metaDIEA (last access 21 September 2021).

## 2. Implementation

### 2.1. Selection of Algorithms to Include in metaDIEA

Although there is a plethora of published methods for DIEA based on RNA-Seq data and most of them are accompanied by the respective software tools, our research indicated that a significant portion of these tools are poorly maintained or documented, are designed to operate under specific non-flexible conditions, or are black-box, closed-source commercial solutions. Therefore, although we identified 25 potential DIEA tools for the purposes of the present study, we posed a series of additional criteria so as to keep only those tools that: (i) are open-source, (ii) have an active user community in the sense of usage and citations, (iii) are relatively user-friendly as non-experts should be able to adequately use them and (iv) are still under active development or support. Notably, out of the 25 identified tools, only 10 were found to still be in active maintenance or development and out of them, a subset of six different tools met all our additional criteria. These tools were sleuth [21], New Tuxedo Suite [22], Tuxedo Suite [23], EBSeq [24], BitSeq [25], and RSEM [26].

As already mentioned, many DIEA tools are not consistent with respect to a common output format including the significance metrics of the results (e.g., *p*-values) and their runtime and execution models. The six selected tools are based on different execution models. Specifically, their runtime environments span several profiles, including Python, R packages and scripts, standalone executable binaries and also combinations of the above wrapped with a runtime script. However, the most important difference between them is that they produce different metrics of evidence and prediction from the underlying differential analysis procedures. For example, some provide for *p*-value and a *q*-value for each prediction (e.g., Tuxedo Suite), some provide the posterior probability of differentially expression (PPDE) (e.g., RSEM) and others provide the probability of positive log ratio (PPLR) (e.g., BitSeq). The difference between the output metrics of all tools, renders them incompatible for comparison since no direct relation exists between the metrics returned by each tool. Hence, because of this heterogeneity of the final outputs, it is virtually impossible to combine all predictions and end up with a weighted meta-prediction such as the combined *p*-value proposed by the initial PANDORA algorithm. Last, when provided with the same input data, the different tools do not reach the same conclusions. Although this is expected to a certain extent, there may be cases of significant deviations.

To overcome the problem of combining and integrating the heterogeneous output of the selected tools, we developed a meta-analysis method that uses ML to compose a final decision on whether a transcript is differentially expressed or not, based on the output of the various different tools. This framework was tested with simulated RNA-Seq data (starting from raw reads) for four discrete species, namely *Homo sapiens*, *Mus musculus*, *Drosophila melanogaster*, and *Arabidopsis thaliana*, where different ML models were trained and evaluated based on the simulated reads. More specifically, for each simulated dataset, all six tools were executed and four ML models (support vector machines, random forest, rotation forest and XGBoost) were trained and evaluated against the ground truth as derived from the simulated data. Once a model is built, it can be used with new data from the same species without the need for re-training or fine-tuning. In the case of another organism, the methodology of training the ML models presented in this article can be replicated based also on the accompanying pipelines and docker containers.

### 2.2. Integrative Differential Isoform Expression Analysis with Machine Learning

In order to effectively combine the outputs of several DIEA algorithms and integrate the outcome of each individual tool towards a joint solution reflecting the advantages of each algorithm as well as mitigating the respective disadvantages, we developed an integration workflow based on established top-performing ML methodologies and tested it on simulated data. More specifically, the ML workflow integrates six separate DIEA workflows centered around the tools sleuth, New Tuxedo suite, the previously developed Tuxedo suite, BitSeq, RSEM and EBSeq. The rationale behind the selection of these tools is further explained in the Materials and Methods section.

**Figure 1:**
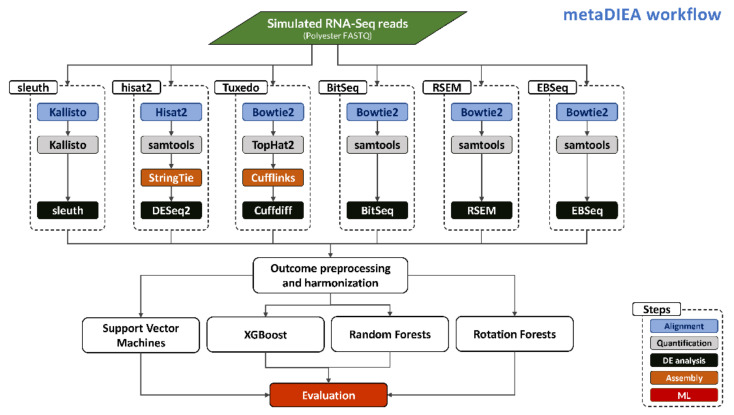
The metaDIEA workflow.

Our approach is depicted in Figure 1 and the process starts with the generation of several replicates of simulated short reads in FASTQ format using the Polyester R package [27]. Subsequently, the FASTQ files are provided to each individual pipeline. The sleuth pipeline performs pseudo-alignment [28] of the RNA-Seq reads and quantification of transcripts with Kallisto, followed by differential expression analysis using sleuth. The New Tuxedo suite pipeline performs full alignment of the reads followed by quantification and transcriptome assembly using samtools and StringTie. The results are interrogated for differential expression using DESeq2. The previous Tuxedo suite performs read alignment and quantification using tophat2 (a wrapper around the bowtie2 aligner that deals with splicing) followed by transcriptome assembly with Cufflinks and a differential expression analysis with Cuffdiff. The BitSeq pipeline deploys bowtie2 for read alignment followed by the usage of samtools for quantification and the BitSeq algorithm for the detection of differentially expressed transcripts. The RSEM pipeline follows a similar path as the BitSeq pipeline, aligning reads with bowtie2 and quantification with samtools, and then differential expression is performed with RSEM or EBSeq. Finally, the EBSeq pipeline is again similar to the previous two regarding alignment and quantification but performs a differential expression analysis with EBSeq.

All the pipelines resulted in lists of candidate differentially expressed transcripts accompanied by the evidence scores reported by each algorithm (see Section 5). As these metrics are often incompatible with each other, several ML methods were deployed to integrate the results. Specifically, the scores of each method were used to construct a vector of features for each transcript. Then, this vector along with the ground truth outcome known from the simulated biological conditions was used to train the ML methods we used. The final assessment of the ML integration was performed as described in Materials and Methods.

## 3. Results

### 3.1. Shiny Implementation

Shiny is an R framework for building interactive web applications purely written in R without the need of writing any additional web related code (e.g., JavaScript, HTML). A Shiny application is a user interface (UI) connected to a server that runs R. The main application script is composed of two separate functions, one containing all the necessary logic the program needs in order to run smoothly and the other containing all user-related features. In addition, Shiny deploys reactivity, i.e., when an input changes, the output changes automatically with minimal developer effort.

A simple application was developed for the purpose of generating RF and XGBoost models automatically. The user can process and visualize the data obtained from the DIEA tools, combine all or a subset of them and train several ML models for the purpose of finding the best hyperparameters that fit the data. The execution time of this grid search varies due to both the number of models specified by the user as well as the number of data combined. Once the most suitable model is found, the user can make predictions about the data related to the levels of DIEA. Two already generated models (one for RF and one for XGBoost) are shipped with the application, based on our analysis, and can be used for prediction without the need of first training user-specified models.

**Figure 2:**
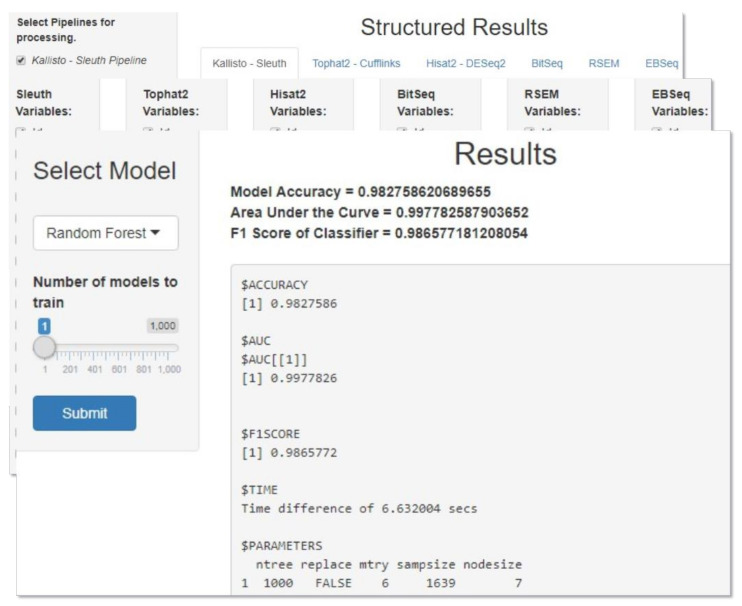
Some notable panels of the Shiny application that implemented the metaDIEA framework. The application supports the execution of predefined RNA-Seq analysis pipelines, the construction of dedicated ensemble models based on either user-provided data or the output of prior executed pipelines, and the review of the produced results.

Upon launching the application, the user is asked to choose the mode in which they want to run the application. Currently, two modes are available, one for training ML models and one for making predictions about the DE level. If the model training mode is picked then the user can specify the tools to be used for processing in the first panel, followed by picking specific variables from each tool in the second one. The third panel in this mode consists of the actual training process where the type of model along with number of models to train can be specified. Once the grid search is over, the best hyperparams and some evaluation metrics are printed on the screen and the generated model can be saved as an .rds file. If the prediction mode is picked, the user is asked to provide the file to be used for prediction in the first panel followed by the model to be used (either an .rds file or one of the already available models) in the second panel. The outcome, i.e., the predicted values, can be saved in a .csv file. A combined snapshot of the metaDIEA Shiny application is depicted in Figure 2.

### 3.2. The metaDIEA Combined Approach Outperforms Individual DIEA Algorithm Performance

In order to assess the overall performance of the metaDIEA combined approach, we evaluated the results produced by each method (individual DIEA and the ensemble metaDIEA approach), across a number of objective criteria. These include five standard metrics utilized in machine learning (i.e., accuracy, sensitivity, specificity, positive predictive value, negative predictive value and area under the curve—see also section performance evaluation metrics in Materials and Methods), as well as three DE-specific characteristics, i.e., the classification of a transcript as DE (regardless of up/down regulation), the true positive classification of an up-regulated transcript and the true positive classification of a down-regulated transcript. These eight metrics, taken together, can provide a robust and objective assessment of the validity of each method towards the identification of DE transcripts, while avoiding overfitting of any given model. In all cases, the ground truth is provided by a simulated dataset, with a priori of defined characteristics.

Regarding the individual algorithms, Table 1 presents the six evaluation metrics for all ten methods for the case of simulated data based on *Mus musculus*. The last row of the table lists the method that produced the best value for the particular metric. Although XGBoost outperforms all others in four out of the six metrics, all methods appear to perform closely. It is quite evident that there is no single method that clearly outperforms all others; as such, the ranking of each method was performed by aggregating the performance of all metrics as an average value, and ranking all methods based on this indicator as shown in Table 2. In this context, the method of XGBoost takes the top position for the detection of a DE transcript, while RF and EBSeq are second best, with the same mean value.

Moreover, our results indicate that in principle, the combined approach using ML methods outperforms in most cases the application of individual algorithms for DIEA. Although not every ML integration outperforms all the single algorithms, XGBoost and RF seem to perform better both in terms of overall ranking (Table 2) as well as capturing more accurately the ground truth as defined by our simulation parameters. Taken together, our results indicate that in general, ML integrations outperform the application of individual algorithm, especially for XGBoost and RF.

Apart from the application of our pipeline to the species *Mus musculus* (mm10), we also performed the same analysis using simulated data for *Homo sapiens* (hg19), fruit fly *Drosophila melanogaster* (dm6), and *Arabidopsis thaliana* (tair10) with the detailed results available as Supplementary Material. Specifically, Tables S1 and S4 depict the performance and ranking respectively for simulated data based on human (*Homo sapiens*, genome version hg19), Tables S2 and S5 depict the performance and ranking for simulated data based on fruit fly (*Drosophila melanogaster*, genome version dm6) and Tables S3 and S6 depict the same metrics simulated data based on the Arabidopsis plant (*Arabidopsis thaliana*, genome version tair10). Overall, similar patterns of performance and ranking are observed, with the ML integrative approaches, especially XGBoost and RF, generally outperforming the individual DIEA pipelines. The only exception is the method ranking in human simulated data, where EBSeq appears to slightly outperform the ML methods when the individual performance metric values are averaged and sorted as a summary performance statistic.

### 3.3. metaDIEA Stability Analysis and Execution Times

**Figure 3:**
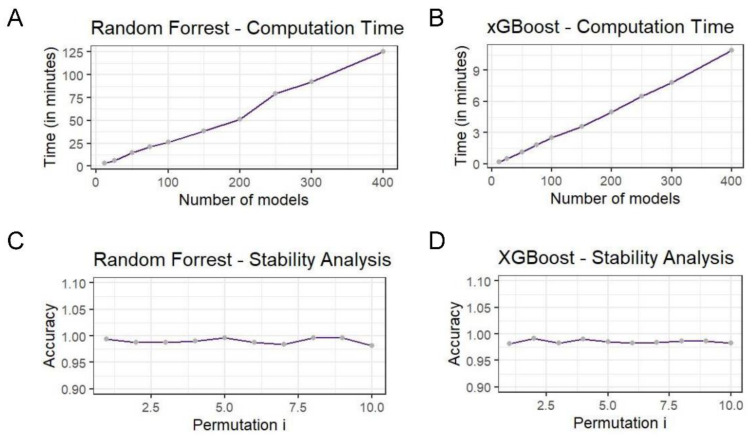
The stability and overall time complexity of the two ensemble methods, random forest and XGBoost. The stability plots (Panel (**C**) for random forest and (**D**) for XGBoost) show the impact in the algorithm accuracy based on various permutations of the training dataset. The time complexity plots (Panel (**A**) for random forest and (**B**) for XGBoost) show the execution time required as a function of the number of models generated in the training process. It is clear that, although both of the ensemble methods are fairly stable and with a linear time complexity, the xGBoost method exhibits a near zero variation in stability and it is significantly faster compared with the random forest approach.

In order to assess the stability of the produced models, a 10-fold cross validation (CV) method was used. The accuracy of the ensemble classifier was picked as a stability metric. During cross validation, the dataset was first randomly perturbed and then split into training and testing sets composed by 9- and 1-fold splits respectively. The training set was fitted to one of the corresponding models (one of RF or XGBoost) and the test data were predicted during the evaluation process. The results are depicted in Figure 3. In the case of random forest, the model accuracy is quite high even when trained with different sets of data, showing how powerful the predictor ensemble solution is. Similar results are available for the XGBoost solution as well and can be found in the  Supplementary Material.

Apart from the stability assessment we measured the execution time of a random grid search for both RF and XGBoost models. During random grid search, a sample combination was picked for training a model each time at random from the expanded grid space of hyperparameters. In this case, 10 executions were measured with a different number of models produced each time. There is evidence for a linear dependence between the number of trained classifiers and the time taken for the search to complete.

## 4. Discussion

Although the application of RNA-Seq protocols for detecting the abundance and differential expression of genes across many species (known model organisms as well as newly sequenced ones), biological conditions and mechanisms such as disease generation and progression has been established for many years now, very frequently researchers remain confused as to which analysis methodology is best suited for own data and the problems under investigation. The perplexity of the landscape is often further increased by the variety of questions that an RNA-Seq dataset can be interrogated for, apart from the standard differential expression analysis, such as alternative splicing detection, de novo transcriptome assembly and calling of variants on RNA-Seq data. As a result of the wellness of applications, several computational methods have been developed to tackle the aforementioned problems. However, a consequence of this wellness is also the scattering and dispersity of these methods across undocumented software libraries, scripts hidden in related articles and packages developed for specific problems and later left unmaintained. Another consequence is the little recorded effort to integrate the multiple existing solution in an effort not only to increase the accuracy of final outcomes by combining the advantages of individual packages but also to maintain the integration result under a common framework and render its application reproducible and documented.

Driven by the aforementioned facts, we previously integrated successfully individual differential expression detection algorithms using a *p*-value combination approach named PANDORA and we showed among others that PANDORA effectively controlled the tradeoff between true positives and false hits [19] and that it is able to offer an analysis framework robust against fluctuations such as the application of different normalization algorithms, leading to a more unbiased downstream analysis [20]. As PANDORA did not treat the case of differential isoform/transcript detection, in the present work we set out to investigate its possible application for DIEA. Through the investigation of this transition process, we found out that the PANDORA *p*-value combination was not suitable for the integration of individual DIEA tools, mostly because of the heterogeneity of the output of each tool. Therefore, we resorted to using established ML techniques to perform the combination of the different tools. Subsequently, our computational experiments using simulated datasets for various organisms suggested that not only the ML approach overall succeed in providing better outcomes than the usage of individual tools, but also provides a framework for applying ML to other similar issues in the future.

To this end, we applied four distinct ML techniques using the outcomes of six established, widely-used and well-maintained pipelines for DIEA. In general, the ML methods presented better results than using a single DIEA method alone in most of our experiments, while two of them, random forest and XGBoost, managed to excel across all our experiments.

Notably the performance of all six methods was consistent across the different species: EBSeq and RSEM were always among the best performing methods. On the other end, New Tuxedo suite and BITSeq were always found in the lowest rankings. Regarding the ML methods, XGBoost and random forest always outperformed all other ML methods and in total most of the times they also outperformed all six methods. The performance of the other two ML methods, i.e., rotation forest and support vector machines were always somewhere in the middle; not the best performance but neither among the last ones.

Similar to the original PANDORA concept for integration of multiple algorithms for differential expression analysis of bulk RNA-Seq data, the metaDIEA integration workflow remains simplistic and intuitive at its fundamentals. While it presents these condensed characteristics, it nevertheless achieves a major computational goal, which is the partial remedy of benchmarking single methods for a given dataset. As already mentioned, the wealth of available DIEA methods often confuses biomedical researchers and even trained bioinformaticians as to which method fits best the particularities of an RNA-Seq dataset. As a result, a lot of valuable research time is spent on benchmarking many algorithms with sometimes completely different syntactic rules, runtime environments and heterogeneous outcomes. On top of that, many times this benchmarking procedure must be repeated for new datasets, losing even more valuable time in repetitive procedures while holding back from spending quality time in answering the original biological questions. MetaDIEA offers an effective framework which integrates many DIEA methods under a single framework while providing evidence regarding the increased performance and accuracy of the results and therefore may comprise a valued research companion for gene expression research across several domains, species and datasets.

Furthermore, because of tremendous and fast-pace technological advances in computation, such as the continuous emergence and development of cloud-computing and the resulting portability demands, there is an increasing trend for containerizing applications and workflows, and bioinformatics [29]. MetaDIEA was developed and tested with compliance and harmonization with this trend in mind. As a result, all the presented workflows are also available as docker containers which may facilitate further the analysis of RNA-Seq data and contribute to timely research results as well as maximize the potential impact and usability of metaDIEA.

## 5. Materials and Methods

### 5.1. Generation of Simulated Datasets

The basis of simulated datasets was simulated RNA-Seq read sets created with the polyester R/Bioconductor package [27]. Polyester allows the simulation of RNA sequencing experiments with differential transcript expression. Given a transcript annotation and coordinates input and arguments specific to the simulation parameterization, it produces simulated read sets. We created simulated read datasets for four discrete species, namely *Homo sapiens* (genome version hg19), *Mus musculus* (genome version mm10), Drosophila Μelanogaster (genome version dm6) and *Arabidopsis thaliana* (genome version TAIR10). More specifically, for each species, three distinct simulated datasets (Set1, Set2, and Set3) with different characteristics regarding the number of differentially expressed transcripts and the number of up and down regulated transcripts were created from a subset of 20,000 transcripts. Set1 consisted of 500, Set2 of 1000, and Set3 of 2000 differentially expressed transcripts. In each set, half the transcripts were up-regulated and the other half down-regulated. For each set six paired-end FASTQ files were produced, three up-regulated and three down-regulated resulting in 18 FASTQ files for each species which comprised the starting points of the downstream processing.

### 5.2. Machine Learning Integration

Because of the heterogeneity of the output of each tool, previous approaches such as *p*-value combination [19,20] could not be applied. However, ML methods are well-known for combining various homogeneous or heterogeneous inputs and reaching a conclusion with limited human intervention. Four ML models were chosen for the needs of the specific work: support vector machines (SVM), random forest (RF), rotation forest (RTF) and XGBoost. SVMs are well-suited for classification and prediction problems and are known to be not very prone to overfitting and resistant to noisy data. RF is an ensemble-based method that offers versatility and ease of use, thus making RF one of the most popular ML methods. RTF is another ensemble-based method that is based on feature extraction. All three methods are supervised ones and produce models that are considered black boxes since it is difficult, if not impossible, to interpret their results. Finally, XGBoost is an ensemble classifier based on the implementation of gradient boosted decision trees. More specifically:

- A support vector machine creates a hypersurface that divides data points plotted in a multidimensional plane, according to their (different) characteristics. The original space is divided into partitions, as homogeneous as possible, by what is called a hyperplane. SVMs are known to model highly complex relationships and have been successfully used in many fields, from text and image recognition [30] to bioinformatics [31]. Moreover, they are easy to understand and apply when used for binary classification, such as in our case of interest, that of DIEA;
- Random forest [32] is a tree-based ensemble classifier, which incorporates the random feature selection in the decision tree creation procedure, providing additional diversity (forests) in the created tree model. Ensemble methods are based on the idea that by combining multiple weaker learners, a stronger learner is created. RF models are preferable to other tree-based classifiers, since they are less prone to over-fitting. However, unlike decision trees, the rationale behind the produced model is not always easy to interpret;
- Rotation forest [33] is another ensemble method classifier introduced relatively recently. It works similarly to random forest; however it has a major difference in the algorithm of internal feature selection. RTF uses a principal component analysis (PCA) in an attempt to choose the more characteristic features and improve the diversity of the forests.
- EXtreme gradient boosting (XGBoost) [34] is an ensemble classifier based on the implementation of gradient boosted decision trees, designed for higher speed and performance. Generally, boosting models sequentially add trees to the ensemble in order to classify correctly the training observations that happened to be misclassified before (without the additional trees). Gradient boosting differs from this by examining the overall classification error, which tries to minimize, by minimizing a specific cost function (taking into account its gradient). XGBoost incorporates this functionality and performs faster and better by making use of additional features such as cache optimization and parallel tree creation.

### 5.3. Analysis Pipeline Implementation

The suggested simulation as well as analysis pipelines were implemented as a series of scripts in Linux shell and the R language. Each script ensures the required dependencies, and all the scripted components are glued together with the help of docker containers. Docker is a software platform that makes use of OS-level virtualization to deliver software applications in packages called containers. For an application to be packed as a container, there exists an initial configuration step which essentially takes place once and is described in a special file called a dockerfile. A dockerfile contains all the necessary software dependencies and instructions for the application to run smoothly. By executing the dockerfile script a docker image is created, which essentially contains the whole application. Dockerfile scripts are divided into layers, rendering the building clearer and more memory-efficient. The application execution is started by initializing a container based on this image. Docker containers are isolated from one another and bundle their own software, library dependencies, and configuration files. They can communicate with each other through well-defined channels.

Therefore, a docker image was created for each one of the selected six DIEA analysis tools. As a base image, for all six images, a lean Ubuntu Linux image of a small size was chosen. By adding software dependencies and installing the necessary packages, all six images were constructed with varying sizes depending on each tool. Once all images are generated, containers based on any of them can be instantiated, thus executing the DIEA analysis offered by each tool.

## 6. Conclusions

Although RNA-Seq revolutionized the study and monitoring of genome-wide gene expression, with a wider dynamic range of measurements and a large portfolio of applications, including the study of expression across single-cells, several challenges such as the golden standards regarding differential isoform expression remain unresolved. On top of that, existing methods are poorly documented or unmaintained. In the present study, we reviewed and evaluated several pipelines for differential isoform expression using simulated data. We proposed an integration framework that combines six individual pipelines using machine learning and provided evidence regarding the performance improvement in the detection of differentially expressed transcripts. The pipelines are available as docker containers for enhanced portability and a Shiny application was also developed to facilitate users of various computational experience levels.

## Figures and Tables

**Table 1:** Performance metrics for the tested DIEA individual algorithms and ML integrations. For each of the six chosen DIEA methods and the four ML approaches, the performance on the six metrics is evaluated based on *Mus musculus* (mm10) simulated data. The last row of the table depicts the winning methodology according to each recorded evaluation metric. XGBoost outperforms all others in four out of the six metrics; however, all methods appear to perform closely.

Method	Accuracy	Sensitivity	Specificity	NPV	PPV	AUC
BitSeq	0.9442	0.5233	0.9984	0.942	0.9771	0.7609
EBSeq	0.9901	0.9733	0.9923	0.9965	0.9421	0.9828
Hisat	0.9472	0.9511	0.9466	0.9934	0.6967	0.9489
XGBoost	0.9928	0.9980	0.9522	0.9837	0.9939	0.9838
RF	0.9807	0.9803	0.9844	0.9845	0.9793	0.9793
RSEM	0.9884	0.9281	0.9961	0.9908	0.9687	0.9621
RTF	0.9807	0.9779	0.9836	0.9831	0.9768	0.9768
Sleuth	0.931	0.5543	0.9795	0.9446	0.7769	0.7669
SVM	0.9782	0.9777	0.9843	0.9832	0.9771	0.9766
TopHat	0.9773	0.9336	0.9829	0.9914	0.8754	0.9582
**Best**	**XGBoost**	**XGBoost**	**BitSeq**	**EBSeq**	**XGBoost**	**XGBoost**

**Table 2:** Ranking of the tested DIEA individual algorithms and ML integrations. For all six methods and four ML approaches on *Mus musculus* (mm10) simulated data, the ranking of each method is presented for each performance metric. In the last column of the table, the ranking of each method is presented by aggregating the performance of all metrics as an average value, and ranking all methods based on this indicator. Based on this empirical metric, XGBoost outperforms RF and EBSeq, which are both placed in second place.

Method	Accuracy	Sensitivity	Specificity	NPV	PPV	AUC	Mean
XGBoost	1	1	9	6	1	1	1.9
RF	4	2	4	5	2	3	2
EBSeq	2	5	3	1	7	2	2
RSEM	3	8	2	4	6	6	2.9
RTF	5	3	6	8	5	4	3.1
SVM	6	4	5	7	4	5	3.1
TopHat	7	7	7	3	8	7	3.9
BitSeq	9	10	1	10	3	10	4.3
Hisat	8	6	10	2	10	8	4.4
Sleuth	10	9	8	9	9	9	5.4

## Data Availability

Not applicable.

## Supplementary Materials

The following are available online at https://www.mdpi.com/article/10.3390/mps4040068/s1, Tables S1–S6 present the performance of each DIEA single and ML integrated method in using simulated data from three more species.
