# Supplementary material for: Combining Multiple RNA-Seq Data Analysis Algorithms Using Machine Learning Improves Differential Isoform Expression Analysis
Source: Methods Protoc. 2021 Sep 27;4(4):68. doi: 10.3390/mps4040068 (PMC8544431; doi:10.3390/mps4040068)
Supplement: Supplementary file 1 [file mps-04-00068-s001.zip › mps-1290406-supplementary.pdf]

Combining multiple RNA-Seq data analysis algorithms using Machine Learning improves differential isoform expression analysis

Alexandros C. Dimopoulos [1,2], Konstantinos Koukoutegos [3], Fotis E. Psomopoulos [3] and Panagiotis Moulos [1,*]

[1] Institute for Fundamental Biomedical Research, Biomedical Sciences Research Center 'Alexander Fleming', Fleming 34, 16672, Vari. Greece; ACD: dimopoulos@fleming.gr, PM: moulos@fleming.gr

[2] Hellenic Naval Academy, Hatzikyriakou Ave., 18539 Piraeus, Greece; adimopoulos@hna.gr

[3] Institute of Applied Biosciences, Centre for Research and Technology Hellas, 6th km Charilaou-Thermis rd, Thermi, Thessaloniki 57001, Greece; KK: konstantinos.koukoutegos@gmail.com, FEP: fpsom@certh.gr

[*] Correspondence: moulos@fleming.gr; Tel.: +30 210 9656310

Supplementary material

Supplementary methods

On the selection of stand-alone tools to integrate

Although the literature on the detection of differentially expressed isoforms and alternative splicing is rich and many stand-alone packages have emerged, nevertheless, many of them are either poorly or not supported at all, or even discontinued after the publication of an article describing their underlying methodology. In addition, there are packages such as DEXSeq[1], which although their intended use implies differential isoform analysis, a more in-depth investigation reveals that DEXSeq detects differential exon usage and requires additional handling for specific cases of alternative isoform detection[2]. Last but not least, not all currently supported packages are user-friendly enough so as to be deployed relatively quickly by non-trained bioinformaticians, as they might present runtime problems which are not errors but require special handling by trained experts to complete their runs and provide meaningful results.

Therefore, we only further considered software packages that are relatively user-friendly as non-experts should be able to adequately use them. In this sense, we evaluated all investigated packages with respect to their relatively easy setup, running and completion without errors, not only for the test data provided by the relative developers but also for different data. Some packages had unmet dependencies not allowing to be compiled, while others run seamlessly only for the specific examples presented in their manual pages; they either didn't start processing at all or they never completed execution successfully for different datasets. If after a fair amount of time and effort we were unable to properly execute a software package, then we excluded it from the integration framework and ignored it for the rest of the analysis.

Differential isoform expression analysis methods

We selected the Tuxedo suite, the New Tuxedo suite, RSEM, EBSeq, BitSeq and Sleuth based on the following criteria: firstly, all selected software packages were open-source and had their source code released under a license. Secondly, their development is still active, i.e. their code has relatively recently been updated in a repository or the software authors provide support or there is an active community, independently or under a wider framework such as Bioconductor. Third, they had a reasonable learning curve and documentation, so usage was made possible from others than the original developers. Last, all selected software packages

[1] https://pubmed.ncbi.nlm.nih.gov/22722343/
[2] https://pubmed.ncbi.nlm.nih.gov/26327458/

were cited by other authors in the respective literature. The following outline briefly the methodological approaches of each package.

- The Tuxedo suite offers a set of tools for analyzing a variety of RNA-Seq data, including short-read mapping, identification of splice junctions, transcript and isoform detection, differential expression, visualizations, and quality control metrics. It uses Tophat2 to align the reads on a genome and then Cufflinks to assemble aligned RNA-Seq reads into transcripts, estimate their abundances, and test for differential expression and regulation of transcriptome. The output result file contains gene and transcript expression level changes with statistics such as Fold Change (FC) in log2 scale, p-values (both raw and corrected for multiple testing) and gene- and transcript-related attributes such as common name and genomic coordinates.
- The New Tuxedo suite includes a similar but distinct set of tools like the Tuxedo suite. HISAT2 aligns RNA-seq reads to a reference genome and discovers transcript splice sites while StringTie assembles the alignments into full and partial transcripts, creating multiple isoforms as necessary and estimating the expression levels of all genes and transcripts. The transcripts and expression levels from StringTie were fed to DESeq2 [30] for differential expression analysis. The final output result file of this method contains transcript expression level changes with statistics such as FC (in log2 scale), p-values and q-values (multiple testing corrected p-values).
- RSEM (RNA-Seq by Expectation Maximization) is an algorithm and software tool for quantifying transcript abundances from RNA-Seq data, with or without an existing reference genome. RSEM is designed to work with reads aligned to transcript sequences and outputs both a) an estimate of the number of fragments that are derived from a given isoform or gene and b) the estimated fraction of transcripts made up by a given isoform or gene. The second measure of abundance is used by a script that internally uses EBSeq for isoform differential expression detection. The final output includes FC of the raw data as well as estimations of the posterior FC of the normalized data, and the transcripts with posterior probability of being differentially expressed (PPDE).
- EBSeq is an algorithm coupled with RSEM, however it can also be executed autonomously, without using the default RSEM scripts but instead fine-tuned EBSeq functions. As expected, it produces the same type of output files as when using the default RSEM scripts. However, the actual results are different.
- BitSeq infers transcript abundance and potential differential expression from sequencing data using a Bayesian approach for the estimation of transcript expression level and offers a differential expression analysis. The implementation of the transcriptome expression estimation and differential expression is written in C++ and Python and is also available as a Bioconductor R package. The final output result file contains the estimation of the Probability of Positive Log Ratio (PPLR) for each transcript along with the FC in log2 scale.
- sleuth is a software for analysing RNA-Seq data, both in transcript- and gene-level that ships with an integrated interactive application for exploratory data analysis. It is compatible with kallisto pseudoaligner and accepts as input transcript abundances that have been quantified with the latter. The output of sleuth contains among other parameters, the p-values and q-values of a transcript being differentially expressed.

Performance evaluation metrics

To evaluate the performance of the six individual methods as well as our combined ML approaches, the following metrics were used:

- Accuracy: the ratio of the correctly classified transcripts to the total number of transcripts.
- Sensitivity: the probability of predicting a transcript as DE when it truly is DE.
- Specificity: the probability of predicting a transcript as non DE when it is truly non-DE.

- Positive Predictive Value (PPV): the probability of a transcript being DE when it is predicted as such.
- Negative Predictive Value (NPV): the probability of a transcript being non-DE when it is predicted as such.
- Area Under the Curve (AUC): the area under the ROC (Receiver Operating Characteristic) curve, created by plotting the true positive rate (TPR) against the false positive rate (FPR).

All the implementations, executions and measurements were performed using in-house R scripts and available ML R libraries, including the packages randomForest for Random Forest, kernlab for Support Vector Machines, rotationforest for Rotation Forest and XGBoost for the respective method.

Supplementary tables

Table S1. For each of the six chosen methods and the four ML approaches, using *Homo sapiens* (hg19) simulated data, the performance on the six metrics is evaluated. In the last row of the table, the name of the best performing method for each metric is presented. XGBoost outperforms all others in four out of the six metrics, but it is quite evident that there is no single method that clearly outperforms all others.

Method	Accuracy	Sensitivity	Specificity	NPV	PPV	AUC
BitSeq	0.9634	0.9291	0.9676	0.9909	0.7826	0.9484
EBSeq	0.9889	0.9743	0.9908	0.9968	0.9297	0.9825
Hisat	0.9232	0.9470	0.9203	0.9928	0.5982	0.9336
XGBoost	0.9932	0.9976	0.9582	0.9798	0.9948	0.9893
RF	0.982	0.9821	0.9855	0.9862	0.9808	0.982
RSEM	0.9881	0.9359	0.9947	0.992	0.9568	0.9653
RTF	0.9804	0.9779	0.9832	0.9838	0.9785	0.9785
Sleuth	0.9376	0.9786	0.9325	0.9971	0.645	0.9555
SVM	0.9788	0.9809	0.9838	0.9847	0.9782	0.9791
TopHat	0.9729	0.9377	0.9773	0.9921	0.8379	0.9575
Best	*XGBoost*	*XGBoost*	*RSEM*	*Sleuth*	*XGBoost*	*XGBoost*

Table S2. For each of the six chosen methods and the four ML approaches, using *Drosophila melanogaster* (dm6) simulated data, the performance on the six metrics is evaluated. In the last row of the table, the name of the best performing method for each metric is presented. XGBoost outperforms all others in four out of the six metrics, but it is quite evident that there is no single method that clearly outperforms all others. Another metric worth mentioning in the very poor performance of BitSeq for the specific species, which does not predict correctly not even one DE isoform

Method	Accuracy	Sensitivity	Specificity	NPV	PPV	AUC
BitSeq	0.8877	0	0.9996	0.8881	0	0.4998
EBSeq	0.9746	0.932	0.98	0.9913	0.8543	0.956
Hisat	0.9062	0.8809	0.9094	0.9838	0.5503	0.8951
XGBoost	0.9862	0.9935	0.9289	0.9473	0.9910	0.9769
RF	0.9634	0.9631	0.9713	0.9714	0.9608	0.9609
RSEM	0.9777	0.9103	0.9862	0.9887	0.8924	0.9482
RTF	0.9628	0.9625	0.9709	0.9705	0.9604	0.9592
Sleuth	0.9318	0.9426	0.9305	0.9923	0.6306	0.9365
SVM	0.962	0.9608	0.9694	0.9704	0.9579	0.9581
TopHat	0.9513	0.8149	0.9685	0.9765	0.7653	0.8917
Best	*XGBoost*	*XGBoost*	*RSEM*	*EBSeq*	*XGBoost*	*XGBoost*

Table S3. For each of the six chosen methods and the four ML approaches, using *Arabidopsis thaliana* (tair10) simulated data, the performance on the six metrics is evaluated. In the last row of the table, the name of the best performing method for each metric is presented. XGBoost outperforms all others in four out of the six metrics, but it is quite evident that there is no single method that clearly outperforms all others.

Method	Accuracy	Sensitivity	Specificity	NPV	PPV	AUC
BitSeq	0.9395	0.4849	0.9988	0.9369	0.9821	0.7419
EBSeq	0.9974	0.9986	0.9972	0.9998	0.9791	0.9979
Hisat	0.9635	0.9827	0.961	0.9977	0.7667	0.9718
XGBoost	0.9991	0.9996	0.9952	0.9971	0.9994	0.9997
RF	0.9973	0.9976	0.9979	0.9981	0.9971	0.9973
RSEM	0.9964	0.976	0.999	0.9969	0.9926	0.9875
RTF	0.9972	0.9968	0.9977	0.998	0.9971	0.9967
Sleuth	0.9524	0.9966	0.9467	0.9995	0.7093	0.9716
SVM	0.9972	0.9972	0.9976	0.9981	0.9966	0.9972
TopHat	0.9901	0.9923	0.9898	0.999	0.927	0.991
Best	*XGBoost*	*XGBoost*	*RSEM*	*EBSeq*	*XGBoost*	*XGBoost*

Table S4. For all six methods and four ML approaches on *Homo sapiens* (hg19) simulated data, the ranking of each method is presented for each metric. In the last column of the table, the ranking of each method is presented by aggregating the performance of all metrics as an average value, and ranking all methods based on this indicator. Based on this new metric, EBSeq outperforms RF and XGBoost, which are very close in second and third place respectively.

Method	Accuracy	Sensitivity	Specificity	NPV	PPV	AUC	Mean
EBSeq	2	6	2	2	6	2	2
RF	4	2	3	7	2	3	2.1
XGBoost	1	1	8	10	1	1	2.2
RSEM	3	9	1	5	5	6	2.9
SVM	6	3	4	8	4	4	2.9
RTF	5	5	5	9	3	5	3.2
TopHat	7	8	6	4	7	7	3.9
Sleuth	9	4	9	1	9	8	4
BitSeq	8	10	7	6	8	9	4.8
Hisat	10	7	10	3	10	10	5

Table S5. For all six methods and four ML approaches on Drosophila melanogaster (dm6) simulated data, the ranking of each method is presented for each metric. In the last column of the table, the ranking of each method is presented by aggregating the performance of all metrics as an average value, and ranking all methods based on this indicator. Based on this new metric, RF outperforms XGBoost and EBSeq, which in second and third place respectively.

Method	Accuracy	Sensitivity	Specificity	NPV	PPV	AUC	Mean
RF	4	2	4	6	2	2	2
XGBoost	1	1	9	9	1	1	2.2
EBSeq	3	6	3	2	6	5	2.5
RSEM	2	7	2	3	5	6	2.5
RTF	5	3	5	7	3	3	2.6
SVM	6	4	6	8	4	4	3.2
Sleuth	8	5	8	1	8	7	3.7
TopHat	7	9	7	5	7	9	4.4
Hisat	9	8	10	4	9	8	4.8
BitSeq	10	10	1	10	10	10	5.1

Table S6. For all six methods and four ML approaches on *Arabidopsis thaliana* (tair10) simulated data, the ranking of each method is presented for each metric. In the last column of the table, the ranking of each method is presented by aggregating the performance of all metrics as an average value, and ranking all methods based on this indicator. Based on this new metric, RF outperforms XGBoost and EBSeq, which in second and third place respectively.

Method	Accuracy	Sensitivity	Specificity	NPV	PPV	AUC	Mean
RF	3	3	3	4	2	3	1.8
XGBoost	1	1	7	8	1	1	1.9
EBSeq	2	2	6	1	7	2	2
RTF	4	5	4	6	3	5	2.7
SVM	5	4	5	5	4	4	2.7
RSEM	6	9	1	9	5	7	3.7
TopHat	7	7	8	3	8	6	3.9
Sleuth	9	6	10	2	10	9	4.6
BitSeq	10	10	2	10	6	10	4.8
Hisat	8	8	9	7	9	8	4.9
